# Erythropoietin Over-Expression Protects against Diet-Induced Obesity in Mice through Increased Fat Oxidation in Muscles

**DOI:** 10.1371/journal.pone.0005894

**Published:** 2009-06-12

**Authors:** Pernille Hojman, Camilla Brolin, Hanne Gissel, Claus Brandt, Bo Zerahn, Bente Klarlund Pedersen, Julie Gehl

**Affiliations:** 1 Centre of Inflammation and Metabolism at the Department of Infectious Diseases, Rigshospitalet, University of Copenhagen, Copenhagen, Denmark; 2 Department of Oncology, Copenhagen University Hospital Herlev, Herlev, Denmark; 3 Department of Physiology and Biophysics, University of Aarhus, Aarhus, Denmark; 4 Department of Clinical Physiology and Nuclear Medicine, Copenhagen University Hospital Herlev, Herlev, Denmark; University of Las Palmas de Gran Canaria, Spain

## Abstract

Erythropoietin can be over-expressed in skeletal muscles by gene electrotransfer, resulting in 100-fold increase in serum EPO and significant increases in haemoglobin levels. Earlier studies have suggested that EPO improves several metabolic parameters when administered to chronically ill kidney patients. Thus we applied the EPO over-expression model to investigate the metabolic effect of EPO in vivo.

At 12 weeks, EPO expression resulted in a 23% weight reduction (P<0.01) in EPO transfected obese mice; thus the mice weighed 21.9±0.8 g (control, normal diet,) 21.9±1.4 g (EPO, normal diet), 35.3±3.3 g (control, high-fat diet) and 28.8±2.6 g (EPO, high-fat diet). Correspondingly, DXA scanning revealed that this was due to a 28% reduction in adipose tissue mass.

The decrease in adipose tissue mass was accompanied by a complete normalisation of fasting insulin levels and glucose tolerance in the high-fat fed mice. EPO expression also induced a 14% increase in muscle volume and a 25% increase in vascularisation of the EPO transfected muscle. Muscle force and stamina were not affected by EPO expression. PCR array analysis revealed that genes involved in lipid metabolism, thermogenesis and inflammation were increased in muscles in response to EPO expression, while genes involved in glucose metabolism were down-regulated. In addition, muscular fat oxidation was increased 1.8-fold in both the EPO transfected and contralateral muscles.

In conclusion, we have shown that EPO when expressed in supra-physiological levels has substantial metabolic effects including protection against diet-induced obesity and normalisation of glucose sensitivity associated with a shift to increased fat metabolism in the muscles.

## Introduction

Erythropoietin (EPO) was introduced for clinical use in 1989 in the treatment of end-stage renal disease (ESRD). These early clinical trials showed that EPO increased exercise capacity and activity levels, improved sleep-wake cycles, increased body warmth, and improved appetite and quality-of-life [1]. Later studies showed that EPO treatment also tended to lower BMI and increase underarm muscular area [2], [3]. In fact Allegra and colleagues showed that EPO treatment lowered BMI, LDL, and apolipoprotein B significantly if the patients did not increase their food intake, while this effect was abrogated if food intake was increased [3]. Furthermore it has been shown that leptin, which is secreted by the adipose tissue in proportion to the fat stores [4], is significantly decreased after 3 to 6 months of EPO treatment [5] suggesting a fat reducing effect of EPO. Other trials with EPO administration to diabetic, chronic renal diseased patients have shown that EPO significantly improves several metabolic parameters including fasting glucose level and insulin sensitivity as measured by euglycaemic hyperinsulinaemic clamp and intravenous glucose tolerance test [6], [7].

To our knowledge, no formal study of EPO administration to obese patients has been undertaken. However in an altitude study, 22 obese male subjects with metabolic syndrome were relocated from 500 to 1700 metres above sea level for 3 weeks [8]. During the sojourn, plasma EPO and reticulocyte count, but not plasma haemoglobin increased significantly, and a decrease in body mass was observed, which was mainly due to a reduction in body fat [8]. The subjects also showed significant improvements in the glycemic parameters such as glucose tolerance and insulin resistance [9].

The aim of the present study was to test the hypothesis that over-expression of EPO in mice fed a high fat diet could a) reduce fat accumulation, b) improve diet-induced insulin resistance, and c) normalise glucose tolerance.

Over-expression of EPO was obtained by electrotransfer of cDNA coding for EPO to the tibialis cranialis (TC) muscle. Transfer by electric pulses is obtained when an external electric field transiently destabilises the membrane rendering the cells accessible for cDNA entry [10]. There are several reports on successful *in vivo* DNA electrotransfer of EPO into skeletal muscles in both mice [11]–[13] and rats [14], [15] with EPO expression and elevations in the haemoglobin levels being detected more than a year after DNA electrotransfer in mice [16]. In addition, it has been shown that EPO expression and thus haemoglobin levels can be tightly regulated through a doxycycline-inducible regulatory system [17]. This model was used in the present study to generate a transient conditional transgenic EPO mouse model.

## Results

### Conditional EPO expression from skeletal muscles after DNA electrotransfer

Transient conditional transgenic EPO mice were generated by transferring 1 µg plasmid encoding EPO under control of the doxycycline-responsive element (TRE), 1 µg of plasmid encoding the reverse tetracycline transactivator (rtTA), and 1 µg of plasmid encoding the tetracycline-dependent silencer (tetS) into the right tibialis cranialis muscle by DNA electrotransfer. After treatment the mice were placed on either a normal chow or a high-fat (60 kcal% fat) diet. Treatment of the transfected mice with doxycycline, which induces expression, resulted in significant increases in Hgb levels, peaking 6 weeks after transfection in both lean (14.7±0.4 mmol/l, P = 3.4*10^−9^, n = 8) and high-fat fed mice (14.2±0.6 mmol/l, P = 1.8*10^−6^, n = 8) (Control: 8.4±0.1 mmol/l). A weekly injection of 10 µg/kg recombinant human EPO (rhEPO), induced similar increases in Hgb levels (lean: 14.0±0.3 mmol/l, P = 8.9*10^−10^, n = 8; high-fat fed: 13.3±0.3 mmol/l, P = 5.7*10^−8^, n = 8, **Fig. S1**). After 12 weeks of weekly EPO injections, the Hgb levels dropped to baseline level, while the Hgb levels in the EPO electrotransferred mice remained elevated. The iron levels in the EPO injected mice were significantly higher than in the EPO transfected mice, indicating a high turn-over of red blood cells in the groups receiving rhEPO (**Fig. S1**).

Serum EPO increased from the basal level of 0.04±0.003 pg/µl to 2.39±0.4 pg/µl (P = 0.033, n = 8) within the first 2 weeks after DNA electrotransfer, equivalent to an increase in mean Hgb from 8.2±0.1 to 11.8±0.2 mmol/L (P = 7.8*10^−7^, n = 8). The corresponding EPO concentration was 1390±117 pg/mg muscle in the transfected muscle, 12±3 pg/mg muscle in the contralateral control muscle, and 1.0±0.1 pg/mg muscle in the muscles of control mice. Thus, the EPO concentration in the transfected muscle was 500 times higher than the level reached systemically.

### EPO expression diminishes fat mass in diet-induced obese mice

**Figure 1:**
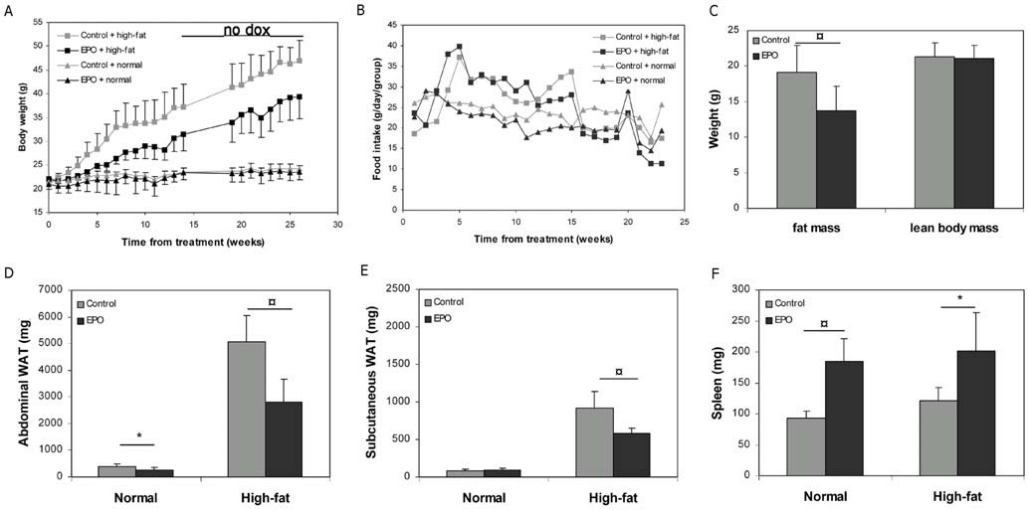
EPO expression diminishes diet-induced fat accumulation. One µg of EPO plasmid and each of the regulatory plasmids, pTet-On and pTetS, were electrotransferred into the right tibialis cranialis muscle of C57Black/C mice. Each group consisted of 8 mice and the mice were placed on either regular chow or a high-fat diet. Means±SEM are depicted. A) Body weight of the EPO transfected mice. For the first 14 weeks transgenic EPO expression was induced by administration of doxycycline in the drinking water, whereafter the doxycycline was withdrawn for the rest of the experiment. B) Food intake for the animals in A). C) Fat mass and lean body mass in the high-fat fed mice measured by DXA scanning 12 weeks after DNA electrotransfer. D–F) Weight of abdominal fat, subcutaneous fat and spleen 12 weeks after DNA electrotransfer. Statistical significance was tested by Student's t-test with Bonferroni corrections for multiple testing. * indicates significance at p<0.05, while ¤ indicates significance at p<0.01.

Mice from both dietary groups were weighed weekly after EPO transfer and induction of gene expression by dox administration. In the high-fat fed groups, EPO expression significantly decreased the weight gain with up to 75% (P = 0.0024, n = 8) in the first 30 days (**Fig. 1A**). In fact, there were no differences in body weight between the EPO transfected high-fat fed mice and mice receiving normal chow (P = 0.23, n = 8). After 40 days, body weight started to increase in the high-fat fed EPO group compared to mice receiving normal chow. This was correlated with a decrease in the Hgb levels from 13.0±1.8 mmol/l to 11.4±3.2 mmol/l, suggesting a reduction in the EPO expression.

Importantly, there were no differences in the food intake in the high-fat fed mice between the control (2.52 g/mouse/day) and EPO transfected groups (2.91 g/mouse/day), or between the control (2.83 g/mouse/day) and EPO transfected groups (3.72 g/mouse/day) placed on a regular diet (**Fig. 1B**).

The high-fat fed EPO transfected mice appeared noticeably leaner than control mice fed the same diet. To assess the body composition of the high-fat fed mice, a DXA scanning was performed 12 weeks after EPO electrotransfer (**Fig. 1C**). The EPO transfected mice had 28% lower amounts of adipose tissue than the control mice (P = 0.012, n = 8), while no difference in lean body mass was observed (P = 0.83, n = 8).

A quantitative analysis of fat-pads and organ masses revealed that high-fat feeding led to significant increases in abdominal and subcutaneous adipose weight (**Fig. 1D–E**). EPO expression in the high-fat fed mice resulted in 44% (P = 0.0004, n = 8) and 37% (P = 0.002, n = 8) reductions in the masses of the abdominal and subcutaneous fat pads, respectively. The reduction in adipose tissue mass was partial in this model as the high-fat fed EPO transfected mice still had appreciably more adipose tissue mass than lean control mice. EPO expression in mice fed a normal chow diet also led to a small, but reproducible decrease in abdominal fat-pad mass (P = 0.021, n = 8). In both EPO transfected groups, the spleens were enlarged due to increased turnover of erythrocytes (**Fig. 1G**). No significant changes in the weights of liver and heart were observed (**Fig. S2**).

### Muscle hypertrophy after EPO electrotransfer

**Figure 2:**
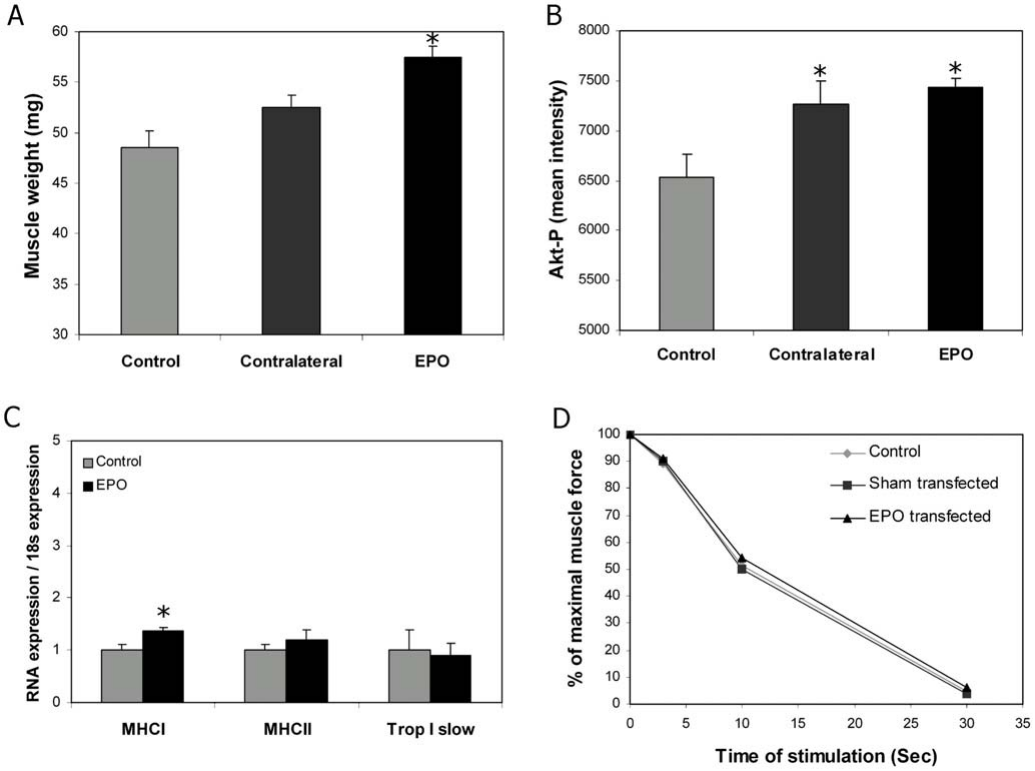
EPO expression is associated with muscle hypertrophy. The muscle hypertrophic response was evaluated 5 weeks after DNA electrotransfer in mice placed on the regular chow diet. Each group consisted of 8 mice. For A and B, data from muscles from control mice (control), the contra lateral leg of the EPO transfected mice (contralateral), and the EPO transfected leg (EPO) are shown. Means±SEM are depicted. A) Total muscle weight, B) phosporylated Akt determined by Western Blotting, C) expression of muscle fibre protein (Q-PCR) in control and EPO transfected muscles, and D) muscle stamina in % of the control leg. Statistical significance was tested by Student's t-test with Bonferroni corrections for multiple testing. * indicates significance at p<0.05.

To examine the phenotypic consequences of EPO expression on the transfected muscle, mice were electrotransferred with EPO in 1 leg and received a chow diet and doxycycline for 5 weeks. As shown in **Fig. 2A**, EPO expression induced significant increases in muscle mass in the transfected muscles compared to the contra lateral control muscle (P = 0.046, n = 8) and muscles from untreated control mice (P = 0.00015, n = 8). The increase in muscle weight tended to correlate positively with the Hgb level (P = 0.078, n = 10). The contra lateral muscles tended to weigh more than the control muscles (P = 0.086). The total protein concentration per g muscle remained unchanged, indicating that the increased muscle volume was due to hypertrophy and not edema formation (data not shown). Electroporation alone did not have any effect on muscle mass (data not shown).

To elucidate the molecular event leading to increased muscle mass, we investigated the level of phosphorylated Akt, which is an important inducer of muscle hypertrophy, and several muscular transcription factors. We found that EPO over-expression significantly increased the phosporylation of Akt (**Fig. 2B**) and induced a 4-fold increase in the expression of myogenin at 5 weeks (data not shown). At this time point, EPO expression did not induce any of the muscular transcription factors, MyoD1, Myf5 and myostatin (data not shown). Despite the increased muscle mass, EPO expression had no effect on maximal force or stamina, which did not differ between electroporated, sham, or EPO transfected muscles (**Fig. 2D**).

Expression analysis of heavy myosin chain (MHC) proteins revealed that MHCI was up-regulated in the EPO transfected muscles (**Fig. 2C**), suggesting that the hypertrophic response might primarily be associated with Type I muscle fiber hypertrophy.

### Increased vascularisation of muscles in EPO transfected mice

**Figure 3:**
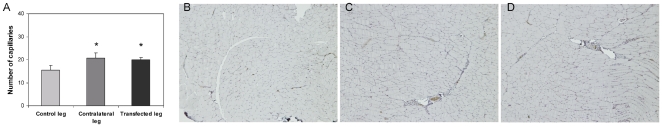
EPO increases vascularisation in the transfected muscle. Histological sections were performed from muscles excised 5 weeks after DNA electrotransfer and stained with CD31. A) Mean number of capillaries per muscle section (n = 8, mean±SEM), and representative pictures of histological sections of B) control muscle, C) contra lateral muscle, and D) EPO transfected muscle. Statistical significance was tested by Student's t-test with Bonferroni corrections for multiple testing. * indicates significance at p<0.05.

In agreement with the reported angiogenic effects of EPO, we found an increase in the number of CD31 positive vessels in both the EPO transfected (20.0±1.0) and the contralateral muscles (20.7±2.3) compared to control muscles from untreated mice (15.5±2.1) (**Fig. 3**). Evaluation of expression of the angiogenic factors, VEGF-1A and VEGF-1B, showed that these were not elevated in the EPO transfected muscles (data not shown).

### EPO expression normalises metabolic parameters in diet-induced obese mice

**Figure 4:**
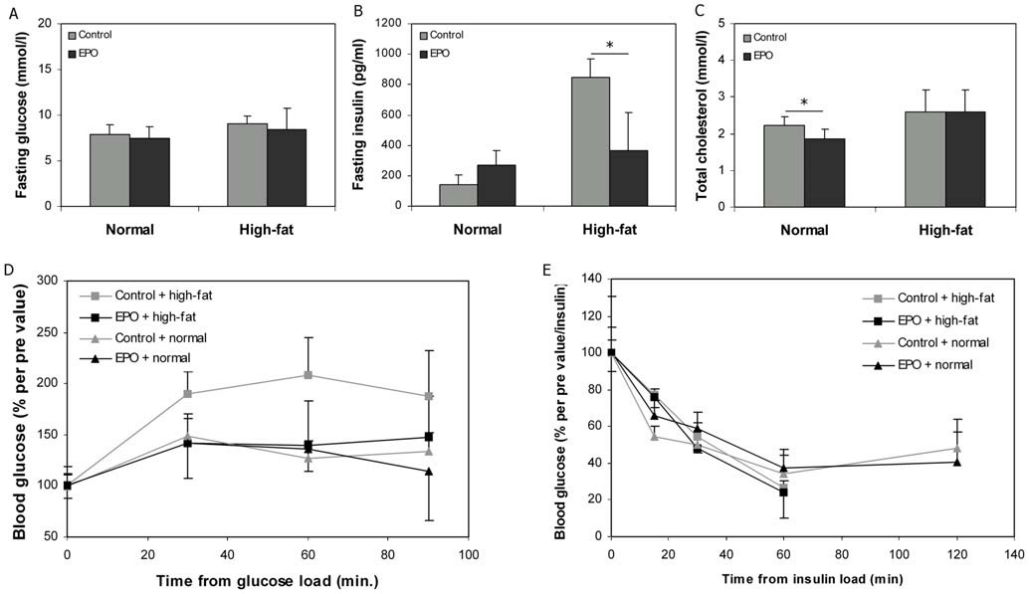
EPO improves glucose tolerance in high-fat fed mice. Twelve weeks after EPO electrotransfer A) fasting glucose, B) fasting insulin, and C) fasting total cholesterol were determined, and D) a glucose tolerance test and E) an insulin tolerance test were performed. Means±SEM for groups of 8 mice placed on either a regular chow or high-fat diet are depicted. Statistical significance was tested by Student's t-test with Bonferroni corrections for multiple testing. * indicates significance at p<0.05.

Next, the effect of transgenic EPO expression on metabolic parameters after 12 weeks of doxycycline induction was examined. There were no differences in fasting glucose between the experimental groups of animals (**Fig. 4A**). Fasting serum insulin was significantly increased in high-fat fed control mice (P = 9.3*10^−8^, n = 7), while insulin levels were normalised by EPO transfection in the high-fat fed mice (P = 0.42, n = 7) compared to normal chow fed control mice (**Fig. 4B**). A reduction in fasting total cholesterol in the chow-fed EPO transfected mice was observed (p = 0.022, n = 8) (**Fig. 4C**), while no differences were observed between the high-fat fed groups. Serum levels of fasting triglycerides, HDL and LDL did not differ significantly between the experimental groups (data not shown).

Glucose and insulin tolerance tests were performed on the different experimental groups (**Fig. 4D–E**). EPO expression in the high-fat fed mice normalised the glucose disposal rate compared to the lean control mice (P = 0.73, n = 8), while no difference in glucose sensitivity was observed between the lean groups. In contrast, neither high-fat feeding nor EPO expression had any effects on insulin sensitivity, although a slower decrease in blood glucose in the EPO transfected mice was observed at 15 min for both dietary groups (P = 0.018 and 0.007, respectively) (**Fig. 4E**). As the mice were injected with insulin per body weight, the high-fat fed mice received more insulin, and therefore also experienced a larger drop in blood glucose. Adjusting for injected insulin, did not results in any significant differences between control and EPO transfected mice. In the high-fat fed groups, the insulin tolerance test was stopped at 60 min as some of the mice developed tremor.

Next, serum levels of selected cytokines and hormones were analysed (**Table 1**). Resistin was normalised in the EPO transfected high-fat group to the level observed in the lean groups. Leptin and tPAI-I levels were increased in both high-fat groups with no effect of EPO expression. TNF-α and IL-6 were not induced to detectable levels in any of the groups (data not shown).

**Table 1:** Serum levels of metabolic hormones.

Parameter	Normal diet			High-fat diet		
	Control	EPO	P-value	Control	EPO	P-value
Leptin (pg/ml)	607±166	699±193	0.71	25384±12703	11766±2890	0.25
MCP-1 (pg/ml)	21.2±2.3	59.6±12.3	0.104	61.3±22.3	72.3±12.2	0.66
Resistin (pg/ml)	4439±1033	3691±670	0.53	8315±1479	3729±528	**0.048**
tPAI-I (pg/ml)	3033±529	2308±410	0.27	8207±2361	7326±883	0.74

Means±SEM of selected hormones in serum after 12 weeks of EPO expression (n = 8 in each group). Statistical significance is tested by Student's T-test with Bonferroni correction for multiple testing.

### Body composition changes caused by transgenic EPO expression are reversible

To examine whether the effects of EPO expression on body mass were reversible, mice were administered doxycycline for 14 weeks, which was then withdrawn for 14 weeks. After 14 weeks of doxycycline withdrawal, Hgb levels had returned to control level for both lean mice (8.0±0.1 mmol/l, n = 8) and high-fat fed mice (8.2±0.2 mmol/l, n = 8). The initial EPO-induced decrease in body weight in the high-fat fed group was reversed after withdrawal of doxycycline, and the weight gain in the EPO transfected group resumed the same rate as the control group (**Fig. 1A**). A glucose tolerance test showed that the normalisation of glucose disposal rate during EPO expression was reversed with the mice exhibiting the same impaired glucose sensitivity as the high-fat fed control group (p = 0.90, n = 8) (**Fig. S3**). No differences in either fasting glucose, or total cholesterol, triglycerides, HDL, or LDL levels were observed between the groups (data not shown).

### Early changes in fasting insulin after EPO expression

In our model the mice develop high levels of Hgb. To circumvent this we investigated the early changes before the Hgb levels increased, as well as the effects of electrotransfer of a mutant form of EPO, which does not cause Hgb to increase.

The early changes were investigated 1 week after EPO electrotransfer. At this point, Hgb levels had increased to around 10.5 mmol/l, and serum EPO concentration was increased 40–fold (**Table 2**). In the high-fat fed EPO transfected mice, fasting insulin levels were significantly reduced (p = 0.012, n = 8), while no differences were observed in fasting glucose levels or glucose sensitivity. No effects on fasting insulin, glucose, or glucose sensitivity were observed in the lean mice.

**Table 2:** Early changes in metabolic parameters one week after EPO electrotransfer.

	Normal diet			High-fat diet		
	Control	EPO	P-value	Control	EPO	P-value
Hgb (mmol/l)	7.6±0.3	10.4±0.5	**0.0002**	8.4±0.2	10.7±0.3	**6.5E-6**
Serum EPO (pg/ml)	52±10	1940±400	**0.0007**	39±9	1664±338	**0.0003**
Fasting glucose (mmol/l)	4.1±0.2	3.7±0.3	0.22	6.2±0.6	5.0±0.3	0.098
Fasting insulin (pg/ml)	847±170	699±233	0.62	1861±337	836±126	**0.012**
Glucose sensitivity (AUC)	1991±51	2076±92	0.44	1459±29	1379±42	0.14

Means±SEM of Hgb, serum EPO, fasting glucose and insulin, and glucose sensitivity one week after EPO electrotransfer (n = 8 in each group). Statistical significance is tested by Student's T-test with Bonferroni correction for multiple testing.

Electrotransfer of EPO mutated at in the strong receptor binding site (aa 46: V→D and aa 50: A→E) resulted in a 36% increase in the muscle mass of the transfected muscle, and a slight reduction in the total body weight 12 weeks after transfection. As evaluated by DXA scanning, there were no changes in the total fat mass in these mice.

### EPO expression induces increased lipid oxidation and inflammatory markers in muscle

To assess the molecular mechanisms by which EPO over-expression diminishes fat mass and normalises metabolic parameters in diet–induced obese mice, PCR arrays on metabolic genes were performed on muscle samples from EPO transfected and control mice from both dietary groups. In addition, C-14 palmitate oxidation was measured directly in muscle homogenates. Analyses were performed at 1 week and 12 weeks after DNA electrotransfer.

**Figure 5:**
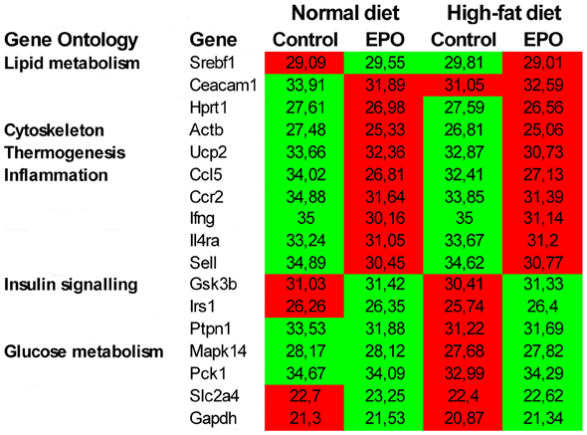
EPO induced changes in muscle gene expression one week after electrotransfer. Significantly differentially expressed genes are depicted according to gene ontology groups. Control and EPO transfected groups were fed either a normal chow or a high-fat diet for one week. Mean Ct threshold is depicted for each group and red corresponds to a high expression value; green indicates a lower expression value (n = 4–5 in each group).

Among the 99 analysed genes, EPO transfection resulted in up-regulation of 12 genes and down-regulation of 15 genes after one week, independent of dietary group. To evaluate the differences between functional related genes a gene ontology analysis was performed (**Fig. 5**). Genes involved in thermogenesis, cytoskeleton and inflammation were up-regulated in response to EPO expression, whereas genes involved in glucose metabolism and transport, proteolysis, stress response, and DNA repair were down-regulated. In agreement with the reduced plasma insulin levels in the high-fat fed EPO mice, genes involved in signalling such as insulin, IRS1 and insulin degrading enzyme were down-regulated in this group. In contrast, genes involved in lipid metabolism, gluconeogenesis, cell-to-cell adhesion, and cell proliferation were up-regulated in the high-fat EPO group.

At 12 weeks, fewer genes were differentially expressed in response to EPO transfection. Three genes (Ccl5, Neurod1, TNF-α), which were all annotated to inflammation, were up-regulated, while 2 genes (PPAR-α, PGC-1α) were down-regulated. EPO attenuated a high-fat feeding-induced increase in several genes e.g. fructose bisphosphatase, forkhead box G1, and Snap23.

**Figure 6:**
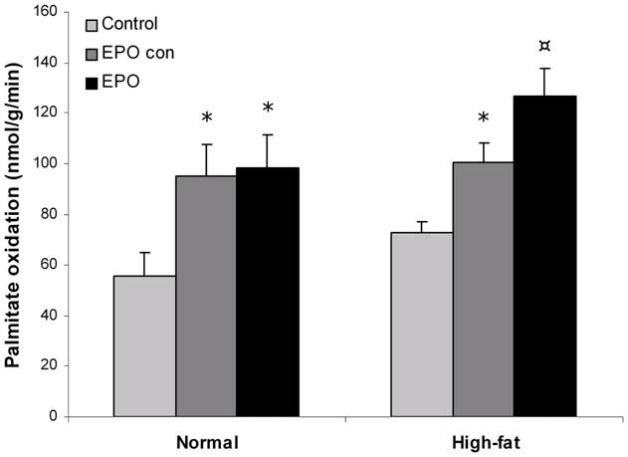
EPO over-expression increases exogenous palmitate oxidation. One week after EPO electrotransfer C-14 palmitate oxidation was measured in muscle homogenates from transfected muscle, the contralateral tibialis cranialis muscle of the same mouse and control muscle from non-transfected animals. Statistical significance was tested by Student's t-test with Bonferroni corrections for multiple testing. * indicates significance at p<0.05, while ¤ indicates significance at p<0.01.

After one week, fat oxidation increased by 64% in the EPO transfected muscles compared to control muscle for both dietary groups (normal: p<0.05, high-fat: p<0.001, n = 8, **Fig. 6**). Similarly, fat oxidation was increased by 63% and 58% in the contralateral muscle of the EPO transfected mice for the normal and high-fat fed groups, respectively (p<0.05 for both groups, **Fig. 6**). The oxidative capacity did not differ between the EPO transfected and contralateral muscles, showing that EPO expression in one muscle increases fat oxidation in the whole musculature.

## Discussion

In the present study, we show that over-expression of EPO protects against diet-induced obesity and improves metabolic parameters in obese mice. These effects were associated with EPO-dependent muscle hypertrophy and increased vascularisation of the muscles. Moreover, EPO expression increased fat oxidation in the muscles and increased the expression of genes involved in lipid metabolism and reduced the expression of genes involved in insulin signalling in the high-fat fed mice, showing that EPO expression shifts the muscular metabolism towards increased fat oxidation.

### EPO over-expression by DNA electrotransfer

We used DNA electrotransfer to over-express EPO selectively in just one muscle i.e. m. tibialis cranialis. In accordance with previous work, EPO electrotransfer to this rather limited amount of tissue was sufficient to induce significant increases in both serum EPO and haematocrit levels [11]–[13]. In our hands, EPO electrotransfer induced the same increase in haematocrit as can be obtained with injections of rhEPO. However the electrotransfer method has the advantage of continuous release of endogenous murine EPO. Although electrotransfer utilises a transient breakdown of the cell membrane, we have used electric pulses, which have been extensively characterised to ensure minimal adverse effects on the muscle tissue and maximal maintenance of muscle survival and function [18]–[20].

### The metabolic role of EPO over-expression

The main finding is that muscular EPO over-expression protects mice against diet-induced obesity. This weight reduction is followed by a normalisation of several metabolic parameters including diet-induced glucose intolerance in the high-fat fed EPO transfected mice. The effects are reversible as increased weight gain and impairment of the metabolic parameters i.e. glucose tolerance are observed after withdrawal of the inducible EPO expression.

Our results show that the mechanism for EPO-induced protection against obesity is through increased fat oxidation as determined directly and by regulation of muscular gene expression, in particular inducing genes involved in lipid metabolism and glyconeogenesis and decreasing glucose metabolism in the high-fat fed mice. In support of this Davenport et al showed that 14 weeks of EPO administration in haemodialysis patients increased muscle glycogen content and lowered muscle fat content [21], while Cayla et al. showed that EPO induces a shift in muscle fibre composition from fast glycolytic fibres to slow oxidative fibres [22]. This suggests that EPO is indeed involved in shifting muscle metabolism towards increasing fat oxidation. Moreover, we also found that EPO induced an increased expression of genes involved in thermogenesis. This correlates well with the findings of Fagher et al., who showed that EPO increases heat production in muscles from haemodialysis patients as measured by direct calorimetry [23]. We have in our current set-up not been able to measure energy expenditure in the EPO transfected mice. Yet the increases in genes involved in thermogenesis in our study suggest that in addition to the increased fat oxidation, increased heat production could play a role in the elevated energy expenditure in the EPO transfected mice.

In the present study, we have measured glucose and insulin tolerance after 12 weeks. At this point the EPO transfected mice had accumulated significantly less adipose tissue than the control mice. EPO expression induced a normalization of glucose tolerance in the high-fat fed mice, while no differences in the insulin sensitivities were observed between the control and EPO transfected mice. The normalization observed in glucose tolerance could most likely be an indication of reduced adipose deposits rather than a direct effect of EPO on glucose disposal rate. This is supported by the down-regulation of genes involved in glucose metabolism, showing that the main effect of EPO most likely to due to the increased overall fat oxidation. The finding that the glucose response to glucose tolerance test is much higher in the high-fat fed controls, also suggests that these have impaired insulin secretion, when challenged with glucose, and that EPO over-expression counteracts this impairment. Our study was not designed to unravel mechanisms underlying altered glucose metabolism. However, although speculative, one explanation could be that EPO influences gut hormones and their effect on insulin secretion.

### EPO over-expression induced muscle hypertrophy and capillarisation

We also show that EPO expression induced muscle hypertrophy as measured by increased muscle weight, while no changes in the amount of protein per muscle weight were observed. The hypertrophic response was most pronounced in the transfected muscle, but we also observed a borderline increase in muscle mass in the contra lateral muscles. Lundby et al. have shown that the EPO receptor is expressed in skeletal muscles in the sarcolemma [24], indicating a physiological role for EPO on muscle tissue. EPO receptor activation leads to stimulation of the signal transducer and activator of transcription (STAT) 5, which activates PI3 kinase and Akt [25]. Akt activation plays an important role in transcription and cell proliferation. We find activation of Akt in both the EPO transfected and contra lateral muscles, suggesting that the hypertrophic response in the muscles is mediated through the STAT-Akt signalling pathway. Interestingly, a transgenic animal model of muscular Akt over-expression showed a similar phenotype as our EPO expressing mice with muscle fibre hypertrophy, resistance to diet-induced obesity and significant improvements in several metabolic variables including glucose tolerance [26]. In our study, we did not find any increases in muscle force. This can be explained by the selective induction of oxidative muscle fibres, which do not specifically add to the maximal contractile force.

The scientific evidence for a role for EPO in promoting angiogenesis in tissues undergoing growth or repair is quite clear [27]. The EPO receptor is located on endothelial cells and cell culture studies with vessels have shown that EPO has direct proliferative effects on endothelial cells [28]. In line with this we find that EPO expression induces increased vascularisation in both the EPO transfected and the contra lateral muscles. The increase in blood supply may play an important role in providing oxygen for the increased oxidative metabolism observed in the muscles.

### Supra-physiological levels of EPO and metabolic effects

Little information on EPO's effect in obese subjects is available due to concern about EPO's role in promoting cardiovascular disease by increasing the haematocrit level and blood pressure in these patients. And in fact we do not believe that our findings are translational to therapeutic applications with native EPO. In our current model, EPO over-expression leads to hematocrit increases even when scaling down the EPO plasmid concentration to 0.25 µg.

New non-haematological EPO agents have been developed primarily for treatment of ischemic stroke [29]. These agents possess all the pleiotropic effects of EPO without raising the haematocrit level. Human EPO consists of a classic four-helix bundle cytokine motif with 2 distinct receptor binding sites. These include 1) a high affinity binding site that involves residues at the helix D:AB loop interface, and 2) a low affinity receptor binding site that lies within the AC helical bundle. The high affinity binding site mediates binding to the homodimer EPO receptor and is responsible for the haematopoietic response, while the low affinity binding site mediates binding to the EPO receptor - common β receptor heterotrimer complex, a interaction, which has been proposed to be responsible for the pleiotropic effects of EPO [30]. We have generated an EPO analogue with mutations in the high affinity binding site for the homodimer EPO receptor, which is responsible for the hematological response. Electrotransfer of this EPO analogue induced muscle hypertrophy and moderate reductions in body weight. However, the EPO analogue was not able to prevent accumulation of adipose tissue. It should be noted that our study used murine EPO, and thus further studies are warranted to fully understand the metabolic sites on both murine and human EPO.

### EPO secretion from the muscle

Recently the skeletal muscle was identified as an endocrine organ that produces and releases a number of biologically active substances, known as myokines [31]. These myokines participate in cell-to-cell and organ-to-organ cross-talk and may be involved in mediating the health beneficial effects of exercise and play important roles in the protection against diseases associated with low-grade inflammation, insulin resistance, and hyperlipidemia such as cardiovascular diseases, type 2 diabetes, and cancer [31].

In our study EPO mimics a myokine in that it is produced and secreted from skeletal muscles and exhibits paracrine and endocrine effects on other muscles. So far no one has been able to show that EPO is secreted from muscles in response to exercise. Contrarily a swimming study showed that serum EPO actually decreased after a training bout [32]. Thus even though we have utilised the DNA electrotransfer method to over-express and secrete EPO from muscles it does not prove that EPO is indeed secreted natively from muscles.

### Conclusion

We have shown that over-expression of EPO protects against diet-induced obesity and this reduction in weight gain was associated with improvement of metabolic parameters in high-fat fed mice in particular increased fat oxidation in the muscles. In addition we found that EPO expression induced muscle hypertrophy and increased capillarisation. Although the strategies used in this study show that haematological and metabolic effects of EPO cannot be separated, they open a new avenue for the development of EPO versions to counteract metabolic syndrome without exacerbated and undesired hematocrit increase.

## Materials and Methods

### Animals and muscle preparation

All animal experiments were conducted in accordance with the recommendations of the European Convention for the Protection of Vertebrate Animals used for Experimentation and after permission from the Danish Animal Experiments Inspectorate. Animal experiments were performed on eight to ten weeks old female C57Black/C mice from Taconic (Tornbjerggaard, Denmark), while force measurements were performed on 4-week-old Wistar rats. The mice were maintained in a thermo-stated environment under a 12-hour light/dark cycle with free access to food and drinking water. The mice were fed either a standard maintenance diet (normal chow, 2844 kcal/kg, 4% crude fat, Altromin pellets, Spezialfutter-Werke, Germany) or a high-fat diet containing 60 kcal% fat (high fat, 5240 kcal/kg, 34.9% crude fat, D12492, Research Diets, Bomholtgaard, Denmark). Food and water were weighed daily and consumption calculated. The animals were killed by cervical dislocation, and intact tibialis cranialis (TC) muscles were excised and quickly frozen on dry ice and absolute alcohol. The subcutaneous fat deposits were dissected by removing the encapsulated fat pad located in the right flank; while the abdominal fat deposits were isolated by insertion into the peritoneal cavity and dissecting out the fat pad associated with the uterus and ovaries. The adipose tissue was then isolated from the non-adipose structures. Moreover, the dorsal fat compartments around the kidneys were cleaned for adipose tissue. Together these compartments constitute the abdominal fat mass.

### Plasmid constructs and in vivo DNA electrotransfer

The plasmids pTet-On, encoding the rtTA transactivator [33], [34], and pTetS, encoding the tS silencer [35], were both obtained from Clontech (Palo Alto, CA, USA). pBI-mEPO encoding murine EPO under the control of an rtTA-dependent promoter was kindly provided by Dr. Fattori. A mutated form of EPO was designed by changing amino acid 46 from V to D and amino acid 50 from A to E, and the construct was cloned into the pBI vector. All DNA preparations were performed using Qiafilter Plasmid Maxiprep kits (Qiagen, Germany), and the concentration and quality of the plasmid preparations were controlled by spectrophotometry and gel electrophoresis.

The animals were anaesthetised 15 min prior to DNA electrotransfer by i.p. injection of Hypnorm (0.4 ml/kg, Janssen Saunderton, Buckinghamshire, UK) and Dormicum (2 mg/kg, Roche, Basel, Switzerland). The plasmid solution (1 µg of each plasmid in 20 µl of PBS) was injected i.m. along the fibres into the TC muscle using an 29G insulin syringe. Plate electrodes with a 4-mm gap were fitted around the hind legs. Good contact between electrode and skin was ensured by hair removal and use of electrode gel. The electric field was applied using a Cliniporator™ (IGEA, Italy), applying a combination of a high voltage (800 V/cm (applied voltage = 320 V), 100 µsec) pulse followed by a low voltage (100 V/cm (applied voltage = 40 V), 400 ms) pulse [18], [20]. Induction of gene expression was obtained by administering drinking water consisting of distilled water containing various concentrations (0.01–0.2 mg/ml) of doxycycline (doxycycline hyclate, Sigma-Aldrich, Denmark) [36]. Control mice were untreated, but received doxycycline in the drinking water.

### Evaluation of body composition by DXA scanning

Twelve weeks after transfection, anaesthetised mice were DXA scanned in a LUNAR Prodigy scanner with software version 8.10 (GE Healthcare Systems, LUNAR, Madison WI, U.S.A.) using the small animal software application. Each group of eight mice was placed closely together in the scanner (in order to reduce the inter-individual variance) and scanned 12 times (to reduce the inter-observational variance). The collected data were analysed by mapping each mouse and extracting individual data for this mouse by local region analysis.

### Protein measurements

Total muscle protein was extracted by tissue homogenisation in a HEPES-EDTA buffer containing 12 mM Na pyrophosphate, 100 mM NaF, NaV and proteinase inhibitors. Protein content was determined by a colorimetric assay (BioRad, Denmark). Equal amounts of protein (25 µg) were separated by a 4–12% SDS PAGE gel (invitrogen) under reducing conditions and then transferred to PVDF membrane (GE healthcare). The membrane was blocked with 5% non-fat milk for one hour at room temperature, and after washing it was incubated over night (4 C°) with either anti-Akt (1∶2000 cell signalling) or anti- phospho-Akt (1∶1000 cell signalling) antibodies in 5% BSA. After washing the membrane was incubated with goat anti rabbit IgG antibodies (1∶10.000 DAKO) in 5% non-fat milk for one hour at room temperature. For detection, the membrane was incubated with super signal west femto luminol/enhancer solution (thermo scientific) for 3 minutes, and the proteins were visualized using a charge-coupled device camera (BioRad) and quantified with Quantity One (Bio Rad).

### Histology

Forty-eight hours after treatment muscles were isolated, fixed in 1 ml formalin buffer, and embedded in paraffin blocks following standard procedures. Sections 3–5 cm thick were stained with hematoxylin and eosin (Mayer-Sour) or with alkaline phosphataseconjugated CD31 antibodies followed by counterstaining with Mayer's hematoxylin.

### Measurement of force

Force was measured as previously described in detail by Clausen & Everts [37]. In brief, isolated EDL muscles were mounted vertically with their tendons intact on a force displacement transducer (Grass FT03, W.Warwick, RI, USA) for isometric contractions in thermostatically controlled (30°C) chambers containing standard Krebs-Ringer bicarbonate buffer (pH 7.3) with (in mM): 122.1 NaCl, 25.1 NaHCO_3_, 2.8 KCl, 1.2 KH_2_PO_4_, 1.2 MgSO_4_, 1.3 CaCl_2_, and 5 D-glucose and gassed continuously with a mixture of 95% O_2_ and 5% CO_2_. Direct electrical stimulation was delivered via platinum electrodes on either side of the mid-portion of the muscle. Muscle length was adjusted to optimal length during repeated stimulation with single pulses. Finally force was checked using short tetanic contractions induced by supramaximal 1 ms pulses of 10 V at 90 Hz for 0.5 sec.

### Blood analyses

Blood samples (20 µl) were drawn monthly from the retro-orbital sinus and haemoglobin levels were measured using the HemoCue Hb201+ (HemoCue AB, Sweden). At termination of the experiments, 500 µl blood was drawn from the mice by cardiac puncture and processed to serum. A Mouse Adipokine Multiplex kit (Millipore Lincoresearch, US) was performed to determine IL-6, insulin, leptin, MCP-1, PAI-I, resistin, and TNF-α serum levels, while serum iron, triglycerides, total cholesterol, and HDL were measured using the Vitros 950 Chemistry System. LDL was calculated from the total cholesterol and HDL levels.

### Glucose and insulin tolerance tests

For each test, the mice were fasted for 16 hours and challenged with 1) an intraperitoneal load of glucose (1 g/kg) for glucose tolerance testing, or 2) an intraperitoneal load of human insulin (0.375 IU/kg, Actrapid, Novo Nordic, Denmark) for insulin tolerance testing. Blood samples (10 µl) were taken retro-orbitally from conscious mice at 0, 30, 60, 90, 120, and 180 min after glucose or insulin load. Blood glucose levels were determined using the HemoCue Glucose 201+ system (HemoCue AB, Sweden).

### Homogenate oxidations

Palmitate oxidation was measured in muscle homogenates using a modified method of that described by Turner et al. [38]. Briefly, muscles were homogenized in 19 volumes of ice-cold 250 mmol/l sucrose, 10 mmol/l Tris-HCl, and 1 mmol/l EDTA, pH 7.4. For assessment of substrate oxidation, 50 µl of muscle homogenate was incubated with 450 µl reaction mixture (pH 7.4). Final concentrations of the reaction mixture were (in mmol/l): 100 sucrose, 80 KCl, 10 Tris-HCl, 5 KH_2_PO_4_, 1 MgCl_2_, 2 malate, 2 ATP, 1 dithiothreitol, 0.2 EDTA, and 0.3% fatty acid–free BSA. Substrates were 0.2 mmol/l [1–14C]palmitate (0.5 µCi) plus 2 mmol/l L-carnitine and 0.05 mmol/l coenzyme A. After 90 min of incubation at 30°C, the reaction was stopped by the addition of 100 µl of 1 mol/l perchloric acid. CO_2_ produced during the incubation was collected in 100 µl of 1 mol/l NaOH. For palmitate incubations, 14C counts present in the acid-soluble fraction were also measured and combined with the CO_2_ values to give the total palmitate oxidation rate

### RT-qPCR analysis

Total RNA was isolated from frozen muscles, livers, and fat pads by tissue homogenisation and RNA extraction using the TRIzol reagent (Invitrogen Life Technologies, Denmark). Mouse diabetes PCR arrays (PAMM023, SABiosciences) containing pre-developed reactions for 90 genes were performed according to the manufacturer's instructions on muscle samples. Data analysis was performed using the web-based analysis tool (www.sabiosciences.com/pcrarraydataanalysis.php), which includes descriptive statistics, including fold change, volcano plots and cluster analyses. For adipose and liver tissues, RT-PCR was performed using random p(dN)_6_ primers (Applied Biosystems, UK) and MultiScripe Reverse Transcriptase (High Capacity cDNA Reverse Transcription Kit, Applied Biosystems, UK). All amplifications were done using the Taqman Universal PCR Master Mix (Applied Biosystems, UK), and sequence specific primers and Taqman probes. PCR and detection were performed using the Taqman 7900 (Applied Biosystems, UK).

## Supporting Information

Figure S1 EPO administration induces large increases in Hgb levels. EPO was administered to groups of 8 mice either by DNA electrotransfer of 1 µg of EPO plasmid and each of the regulatory plasmids, pTet-On and pTetS into the right tibialis cranialis muscle; or by intraperitoneal injections of 10 µg/kg rhEPO. The mice were placed on either regular chow or a high-fat diet and means±SEM for each groups are depicted. A) Time course of hemoglobin levels, and B) serum iron contents measured 12 weeks after start of treatment. Statistical significance was tested by Student's t-test with Bonferroni corrections for multiple testing. * indicates significance at p<0.05, while ¤ indicates significance at p<0.01.
(1.18 MB JPG)

Figure S2 Weight of liver and heart. Twelve weeks after DNA electrotransfer livers and hearts from EPO transfected mice were weighed, and means±SEM for groups of 8 mice are shown. Statistical significance was tested by Student's t-test with Bonferroni corrections for multiple testing, and there were no significant difference between the groups.
(0.68 MB JPG)

Figure S3 The improvement in glucose tolerance is reversed when EPO expression is withdrawn. Fourteen weeks after doxycycline withdrawal in EPO transfected mice, a glucose tolerance test was performed. There was no difference in the glucose tolerance between the control and EPO transfected high-fat fed mice.
(1.49 MB TIF)
