# Supplementary figures and images for: Erythropoietin Over-Expression Protects against Diet-Induced Obesity in Mice through Increased Fat Oxidation in Muscles
Source: PLoS One. 2009 Jun 12;4(6):e5894. doi: 10.1371/journal.pone.0005894 (PMC2690401; doi:10.1371/journal.pone.0005894)

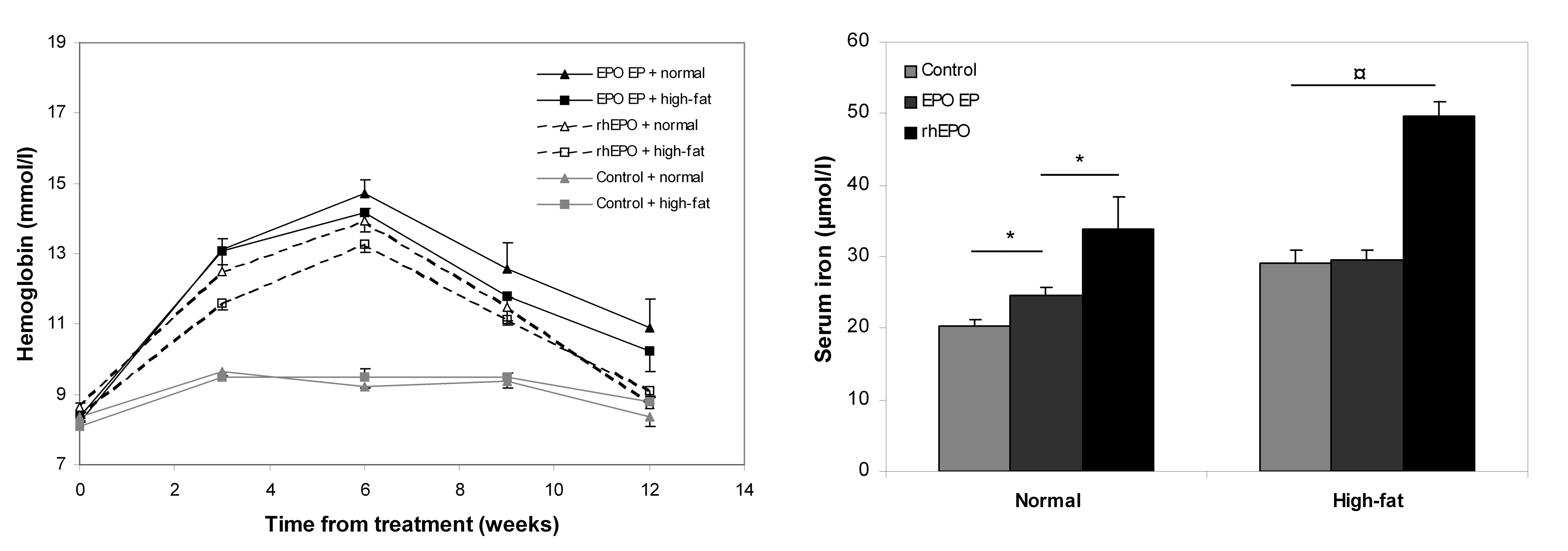

Supplement: Figure S1 — EPO administration induces large increases in Hgb levels. EPO was administered to groups of 8 mice either by DNA electrotransfer of 1 µg of EPO plasmid and each of the regulatory plasmids, pTet-On and pTetS into the right tibialis cranialis muscle; or by intraperitoneal injections of 10 µg/kg rhEPO. The mice were placed on either regular chow or a high-fat diet and means±SEM for each groups are depicted. A) Time course of hemoglobin levels, and B) serum iron contents measured 12 weeks after start of treatment. Statistical significance was tested by Student's t-test with Bonferroni corrections for multiple testing. * indicates significance at p<0.05, while ¤ indicates significance at p<0.01. (1.18 MB JPG) [file pone.0005894.s001.jpg]

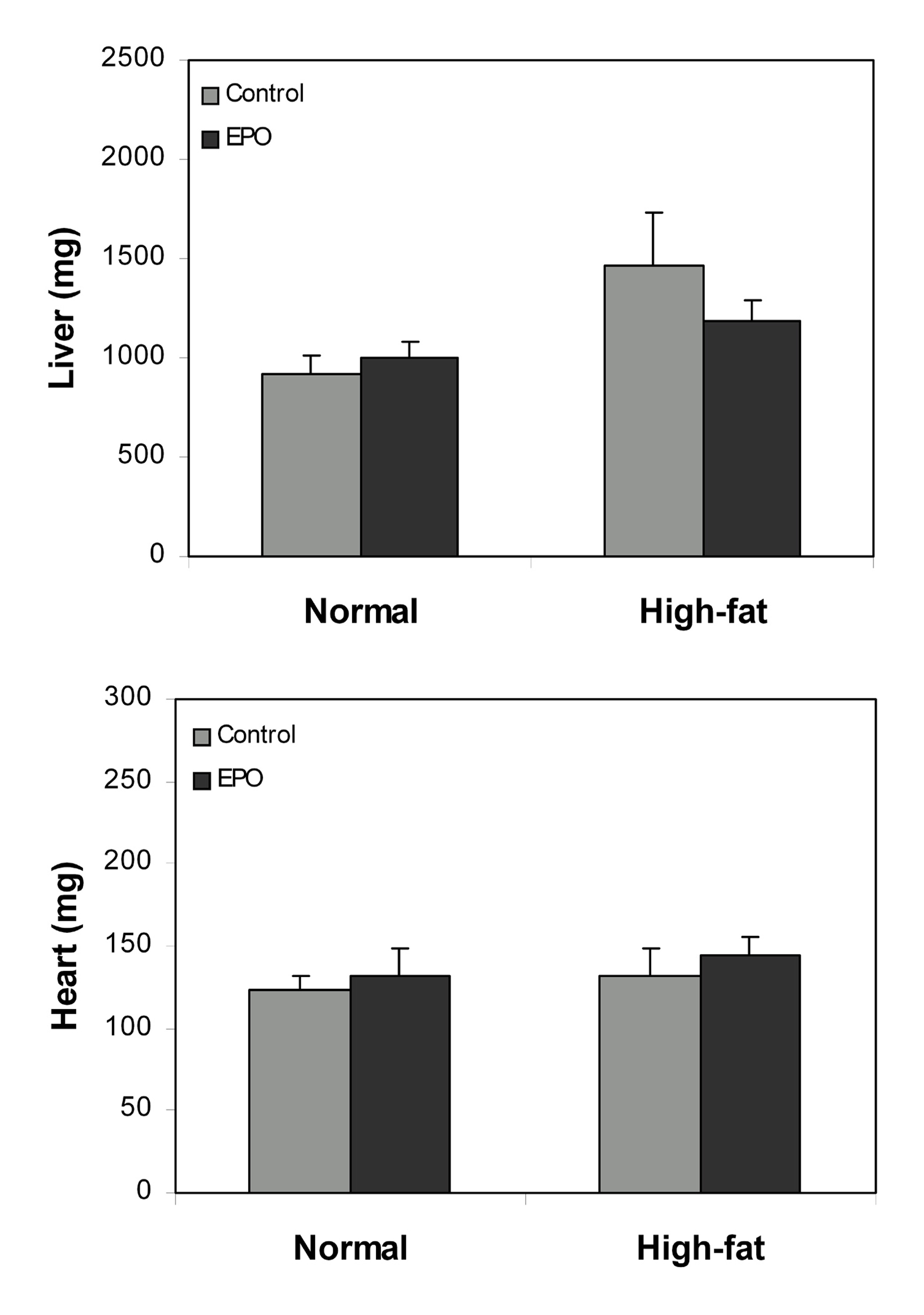

Supplement: Figure S2 — Weight of liver and heart. Twelve weeks after DNA electrotransfer livers and hearts from EPO transfected mice were weighed, and means±SEM for groups of 8 mice are shown. Statistical significance was tested by Student's t-test with Bonferroni corrections for multiple testing, and there were no significant difference between the groups. (0.68 MB JPG) [file pone.0005894.s002.jpg]
